# A Maize Germplasm Is Resistant to Fall Armyworm: From Life Table Analysis to Greenhouse Assays

**DOI:** 10.3390/insects17090897

**Published:** 2026-08-27

**Authors:** Lingling Li, Peiying Li, Wenmeng Li, Changgeng Dai, Hongbo Li

**Affiliations:** 1 Institute of Plant Protection, Guizhou Academy of Agricultural Sciences, Guiyang 550006, China; lilingling2019@163.com (L.L.); is_peiying@163.com (P.L.); lwmhbdgh1028@163.com (W.L.); ggyydai@163.com (C.D.); 2 Guizhou Key Laboratory of Agricultural Biosecurity, Guiyang 550006, China; 3 Guizhou Branch of State Key Laboratory for Biology of Plant Diseases and Insect Pests, Guiyang 550006, China; 4 Key Laboratory of Forest Disaster Warning and Control in Yunnan Province, College of Forestry, Southwest Forestry University, Kunming 650224, China; 5 Key Laboratory of Crop Genetic Resources and Germplasm Innovation in Karst Region, Ministry of Agriculture and Rural Affairs, Guiyang 550006, China

**Keywords:** *Spodoptera frugiperda*, host plant resistance, life table, maize germplasm, feeding preference, oviposition

## Abstract

The fall armyworm (FAW), *Spodoptera frugiperda* (J.E. Smith) (Lepidoptera: Noctuidae), is a devastating pest of maize, and host plant resistance offers a sustainable alternative to chemical control. This study evaluated the resistance of the maize germplasm CH1 against FAW through life table parameters, feeding and oviposition preferences and greenhouse experiment. Results showed that FAW larvae reared on CH1 exhibited significantly prolonged larval development (21.61 d vs. 12.49 d), reduced survival (65.00% vs. 94.00%), and lower fecundity (689.00 vs. 1369.93 eggs per female) compared to those on the susceptible control Huang C. Population parameters, including the intrinsic rate of increase (*r*) and net reproductive rate (*R*_0_), were significantly lower on CH1. Behavioral assays revealed strong feeding and oviposition preferences for Huang C over CH1. Greenhouse experiment confirmed that CH1 plants sustained only minor damage (Davis scale grade 2, resistant), whereas Huang C plants were severely damaged (grade 7, susceptible). These findings demonstrate that CH1 possesses multi-dimensional resistance against FAW, providing a promising genetic resource for breeding FAW-resistant maize varieties and supporting sustainable pest management strategies.

## Simple Summary

The fall armyworm is a destructive pest that causes major losses to maize crops worldwide. Crop insect resistance has become an important component of pest management. This study investigated whether a maize line called CH1 could naturally resist this pest. We compared the growth, survival, and reproduction of fall armyworm larvae fed on CH1 with those fed on a susceptible maize line, Huang C. The results showed that larvae fed on CH1 developed more slowly, had lower survival rates, and produced fewer offspring. In addition, both the larvae and adult moths strongly preferred Huang C over CH1 for feeding and egg-laying. When tested in a greenhouse, CH1 plants showed only minor damage, while Huang C plants were severely defoliated. These findings demonstrate that CH1 possesses multiple layers of natural resistance against fall armyworm. This maize line could serve as a valuable genetic resource for breeding new varieties with built-in pest resistance, reducing the need for pesticides and supporting more sustainable and environmentally friendly farming practices.

## 1. Introduction

Maize, *Zea mays* L. (Poales: Poaceae), is one of the world’s most important crops for grains, feed, and industrial raw material production, playing an irreplaceable role in ensuring global food security and stabilizing agricultural production [1]. The Food and Agriculture Organization (FAO) projected global corn production at approximately 1.217 billion tonnes in 2024 [2]. China is the world‘s largest maize producer. According to the National Bureau of Statistics of China, China’s maize planting area reached 44.74 million hectares in 2024, with a total output of 294.92 million tonnes [3]. Given the immense scale of global and Chinese maize production, yield losses caused by pests have profound implications for food security, farmer livelihoods, and national economies [4].

The fall armyworm (FAW), *Spodoptera frugiperda* (J. E. Smith) (Lepidoptera: Noctuidae), is a key pest causing significant yield losses and quality reduction in maize [5]. Native to tropical and subtropical regions of the Americas, FAW is a major migratory pest that has rapidly spread to many parts of the world in recent years [6]. In China, FAW has become the most important insect pest on maize since its first invasion in Yunnan Province in 2018, posing a serious threat to maize production [7]. The severe damage of maize caused by FAW is largely attributed to its remarkable life history traits, including high fecundity, rapid development, and short generation time [8]. This pest does not undergo diapause and can reproduce year-round under favorable temperature conditions, resulting in multiple overlapping generations per year. Specifically, the egg stage lasts only 2–3 days under optimal conditions [9]. The larval stage typically comprises six instars, with developmental duration varying inversely with temperature, ranging from 9.11 days to 87.42 days [10]. The pupal stage lasts approximately 6.06–20.91 days [10]. In terms of reproduction, a single female can lay over one thousand eggs [8]. These life history characteristics enable FAW populations to expand rapidly within a short period, inflicting substantial damage on host crops.

Currently, FAW management still heavily relies on insecticides and transgenic plants technologies. However, the prolonged and irrational use of insecticides has not only increased pest resistance but also raised concerns about environmental pollution and pesticide residues [11]. Meanwhile, the widespread cultivation of *Bt* maize has exerted strong selection pressure, leading to the development of resistance in FAW field populations to key Bt proteins such as Cry1F and Cry1Ab [12,13], thereby challenging the sustainability of this core control technology. Crop insect resistance has become an important component of pest management, as it can reduce crop damage at the source by affecting pest growth, development, reproduction, or repelling pest feeding, without relying on pesticides or transgenic plants [14]. Consequently, exploring and utilizing inherent host plant resistance of maize is considered one of the most economical, effective, and environmentally friendly strategies for sustainable management of FAW [15]. Landrace germplasm and specific maize accessions, with their rich genetic diversity, serve as valuable resources for discovering resistance genes and breeding resistant varieties [16].

In the Americas, maize varieties with native genetic resistance to FAW, including Perola, Zapalote Chico 2451F, and GEMS-0100, have been developed and widely planted [17,18,19]. Additionally, a series of FAW-resistant maize germplasms, such as the CML lines CML370, CML372, and CML574 have been identified by the International Maize and Wheat Improvement Center (CIMMYT) [15]. Similarly, several resistant temperate maize inbred lines (e.g., Mp496, Mp701–Mp708, Mp713, Mp714, and Mp716), developed by the United States Department of Agriculture’s Agricultural Research Service (USDA-ARS), show relatively high resistance to FAW [20]. In general, resistant host plants significantly influence various aspects of insects, including growth, development, reproduction, physiology, and behavior [21,22]. The life table of an insect provides a detailed summary of survival and reproduction rates across developmental stages [23], offering valuable insights into its potential for population growth in specific environments [24]. In previous studies, life table parameters have been used to evaluate the adaptability of FAW on various resistant maize plants [25]. For example, FAW fed on the resistant maize variety CML442 exhibited lower intrinsic rate of increase (r), finite rate of increase (λ), net reproductive rate (R_0_), gross reproductive rate (GRR), and a longer mean generation time (T), compared to those fed on non-resistant maize [25]. Similar results have been reported, with FAW showing longer developmental duration on the resistant variety Mp708 than on the susceptible one [26]. In China, only one maize inbred line, Xi502, has been found to exhibit moderate resistance to FAW through transcriptomic and metabolomic analyses [15,27]. However, the Xi502 study focused primarily on molecular mechanisms and did not comprehensively evaluate the full spectrum of resistance phenotypes including effects on FAW life history traits, behavioral preferences, or overall plant damage [27]. Therefore, it remains urgent to identify additional maize germplasm resources with multi-dimensional resistance to FAW that can be readily deployed in breeding.

In preliminary screening experiments, 38 maize germplasms were used to evaluate resistance to FAW (unpublished data) and revealed that a maize germplasm CH1 was less infested by FAW. In this study, we used the age-stage, two-sex life table method to analyze the population growth parameters of FAW fed on two maize germplasms CH1 and Huang C (as control). Choice and no-choice assays were then conducted to determine FAW feeding and oviposition preferences. Finally, the resistance of the two maize germplasms against FAW larvae was evaluated under greenhouse conditions. By integrating population, behavioral, and greenhouse experiment data, our study not only demonstrates that CH1 is highly resistant to FAW but also provides a promising resistance source for breeding new FAW-resistant maize varieties.

## 2. Materials and Methods

### 2.1. Insect

FAW individuals were originally collected from maize fields at the Guizhou Academy of Agricultural Sciences, Guiyang, Guizhou province, China (26°51′ N, 106°42′ E) in 2022. A founder population of approximately 200–300 larvae was established and maintained in the laboratory under the following conditions: 26 ± 1 °C, 60–70% relative humidity, and a photoperiod of 16L:8D. The laboratory colony was maintained at a population size of 1000 individuals per generation to avoid inbreeding depression. FAW larvae were reared individually on artificial diet in plastic cups (3.6 cm in height, 5 cm in upper diameter and 3.1 cm in lower diameter). After emergence, FAW adults were paired and transferred to a plastic bag (35 cm in length and 25 cm in width) and provided with 10% (*w*/*v*) honey-soaked cotton ball. Before experiment, FAW was continuously reared at least four generations under above conditions.

### 2.2. Plants

Based on our preliminary screening experiments of 38 maize germplasms for FAW resistance (unpublished data), a maize germplasm CH1 was used to test its resistance to FAW and a maize variety Huang C was used as control. These seeds were provided by Guizhou Drought Grain Sorghum Research Institute, China. Seeds were sown in plastic pots (10.5 cm in height, 12.7 cm in upper diameter and 9.5 cm in lower diameter), with a 1:1 mixture of nutrient soil and vermiculite. Fresh leaves were selected when plants reached the BBCH growth stage 14–15 (approximately 4–5 fully expanded leaves) for the following experiments.

### 2.3. Biological Parameter of FAW Reared on Two Maize Plants

#### 2.3.1. Larval Development Duration and Survival Rate

To investigate the effect of the maize germplasm CH1 on the growth and survival of FAW, newly hatched first-instar larvae were placed individually in plastic cups (3.6 cm in height, 5 cm in upper diameter and 3.1 cm in lower diameter) and fed with leaf sections (3 cm × 3 cm) of CH1, and the survival state of single larvae was recorded every day until pupation. The leaf sections were replaced regularly every 24 h. The FAW larvae reared on Huang C was used as control. Each treatment contained 100 newly hatched larvae.

#### 2.3.2. Pupal Period and Emergence Rate

After pupation, 3-day-old pupae were sexed according to the morphological characteristics of the abdominal end and weighed using an electronic balance (ME204E/02; Mettler Toledo, Shanghai, China). Meanwhile, the survival status of each pupa was observed and recorded daily until adult emergence.

#### 2.3.3. Adult Lifespan and Egg Production

Upon emergence, one pair of adults (one male and one female) derived from the same maize host was placed in a rearing bag (35 cm in length × 25 cm in width) and provided with a 10% honey-soaked cotton ball as a food source. The bag served as both the rearing chamber and the oviposition substrate; eggs were deposited on the inner surface of the bag. The number of eggs on the entire inner surface of the bag was observed and counted daily. To avoid repeated counting of the same eggs on subsequent days, all egg masses were carefully removed from the surface of the bag using a fine brush, dispersed into single eggs under a stereomicroscope (Nikon SMZ800N; Nikon Corporation, Tokyo, Japan), and counted individually. The honey-soaked cotton ball was replaced daily to prevent desiccation and contamination. Upon the initiation of egg-laying, the bag was changed daily until the female adult died. If the male adult died before the female, a replacement male of the same age (derived from the same host treatment) was provided immediately to ensure continued mating opportunities. Each treatment included 30 pairs of adults (30 biological replicates).

#### 2.3.4. Analysis of the Age-Stage, Two-Sex Life Table

Based on the age-stage, two-sex life table theory, raw data were analyzed by the TWO-SEX-MSChart program [28]. The parameters of the age-stage two-sex life tables were calculated according to the following equations [29].

The age-stage-specific survival rate (*s_xj_*) represents the probability that a newly laid egg will survive to age *x* and stage *j*:

$$S_{xj}=\frac{n_{xj}}{n01}$$

The age-specific survival rate (*l_x_*) represents the probability that a newborn egg will survive to age *x*:

$$l_{x}=\sum_{j=1}^{m}s_{xj}$$

The female age-stage-specific fecundity (*f_xj_*) represents daily number of eggs produced by a female at age *x* and stage *j*.

The age-specific fecundity (*m_x_*) represents the number of eggs per individual at age *x*:

$$m_{x}=\frac{\sum_{j=1}^{m}{s_{xj}f}_{xj}}{\sum_{j=1}^{m}s_{xj}}$$

The net reproductive rate (*R*_0_) was calculated as:

$$R_{0}=\sum_{x=0}^{\infty }l_{x}m_{x}$$

The intrinsic rate of increase (*r*) was calculated as:

$$\sum_{x=0}^{\infty }e^{-r(x+1)}l_{x}m_{x}=1$$

The finite rate of increase (*λ*) was calculated as:

$$\lambda =e^{r}$$

The mean generation time (*T*) as calculated as:

$$T=\frac{lnR_{0}}{r}$$

### 2.4. FAW Oviposition and Feeding Preference on Two Maize Plants

#### 2.4.1. Feeding Preference

To evaluate the feeding preference of FAW between Huang C and CH1, we carried out choice and no-choice experiments using the leaf disk method. Leaves of two maize germplasms were cut into square leaf disks with equal area (1.0 cm × 1.0 cm). In the choice test, leaf disks of Huang C and CH1 were respectively placed on two opposite sides of a Petri dish (90 mm in diameter), with equal distances from the center. In the no-choice test, one maize disk from Huang C or CH1 was placed in the center of the Petri dish. The bottom of the Petri dish was coated with 1% agar to maintain moisture. For both tests, newly hatched (first-instar) and third-instar larvae were collected and deprived of food for 3 h, and then transferred to Petri dishes individually using a soft bristle brush. After 24 h for the choice test, the feeding selection was recorded as the number of larvae that fed on each disk. For the no-choice test, the consumed leaf area by FAW was photographed and measured using ImageJ software (ImageJ 1.53q; National Institutes of Health, Bethesda, MD, USA) (in mm^2^). Each treatment contained 10 replications (one larva per Petri dish).

#### 2.4.2. Oviposition Preference

To determine the oviposition preference of FAW between Huang C and CH1, choice test and no-choice test were also conducted. In the choice test, one potted plant of Huang C and one potted plant of CH1 were placed together in a nylon cage (length × width × height = 45 cm × 45 cm × 45 cm), with the pots separated by 30 cm. In the no-choice test, one potted plant of Huang C or CH1 was placed individually in a nylon cage of the same size. For both tests, one pair of virgin male and female adults that emerged for 1 d were placed in each cage to ensure that all eggs laid were fertilized and attributable to the experimental female. The number of eggs laid on the maize plants and on the inner walls of the cages was recorded daily. To avoid repeated counting of the same eggs on subsequent days, all egg masses were carefully removed from the plants and cage walls using a fine brush, dispersed into single eggs under a stereomicroscope, and counted individually. The mean daily fecundity (total eggs per female per day) was subsequently calculated. The positions of the two plants in the choice test were rotated daily to avoid positional bias. Each treatment contained 10 replications.

### 2.5. Resistance Evaluation of Two Corn Plants Against FAW

In June 2023, a greenhouse experiment was conducted at Guizhou Academy of Agricultural Sciences, China (26°51′ N, 106°42′ E), to evaluate the insect resistance of the two kinds of corn plants, Huang C and CH1. The greenhouse conditions were maintained at 28 ± 3 °C during the day and 22 ± 2 °C at night, with relative humidity of 65–85% and natural photoperiod. The potting substrate consisted of a 1:1 (*v*/*v*) mixture of nutrient soil and vermiculite, and plants were irrigated as needed to maintain optimal growth conditions. Seeds of each variety were individually sown in plastic pots (10.5 cm in height, 12.7 cm in upper diameter and 9.5 cm in lower diameter). When the maize grew up to the V3 stage, 15 newly hatched larvae were inoculated into the whorl of each plant. Each germplasm consisted of 10 biological replicates (one plant per replicate, arranged in a completely randomized design). The leaf damage was assessed for each plant at the V6 stage using the modified Davis scale method [30,31]. The resistance level of each germplasm was determined according to previously established criteria with scores of 1 classified as highly resistant (HR), 2–3 classified as resistant (R), 4–5 as partially resistant (PR), 6–7 as susceptible (S), and 8–9 as highly susceptible (HS) [31].

### 2.6. Data Analyses

All statistical analyses were performed using MATLAB (R2024a; The MathWorks, Inc., Natick, MA, USA) and the TWO-SEX-MSChart program [32]. Results are presented as mean ± standard error (SE).

For developmental parameters (developmental duration, pupal weight, adult longevity, and fecundity) and behavioral preference data, normality and homogeneity of variance were first tested using the Shapiro–Wilk test and Levene’s test, respectively. Data that met the assumptions were analyzed using Student’s *t*-test; otherwise, the non-parametric Mann–Whitney U test was applied.

For age-stage, two-sex life table parameters (*r*, *λ*, *R*_0_, and *T*), means and standard errors were estimated using the bootstrap method with 100,000 resampling iterations. Statistical comparisons between treatments were performed using the paired bootstrap test based on 100,000 resamples, as implemented in the TWO-SEX-MSChart program, following the recommendations of Chi et al. [32].

## 3. Results

### 3.1. Development and Reproduction of FAW on Two Maize Plants

As shown in Table 1, no significant differences were observed in the egg duration, pupal duration, or pupal weight of FAW fed on the two maize leaves. However, FAW larvae fed on CH1 exhibited significantly prolonged larval and pre-pupal period and shortened adult lifespan as compared to those fed on Huang C. In addition, the two maize germplasms also influenced the longevity and reproduction of FAW adults. The longevity and oviposition duration of female adults fed on CH1 were shorter, but the pre-oviposition and total pre-oviposition periods of female adults derived from larvae reared on CH1 were longer than those reared on Huang C. Also, the number of eggs laid by adults derived from CH1 was significantly lower than that laid by adults fed on Huang C. However, no significant difference was found in the longevity of male adults derived from the two kinds of corn plants.

### 3.2. Survival Rate and Fecundity of FAW on Two Maize Plants

**Figure 1:**
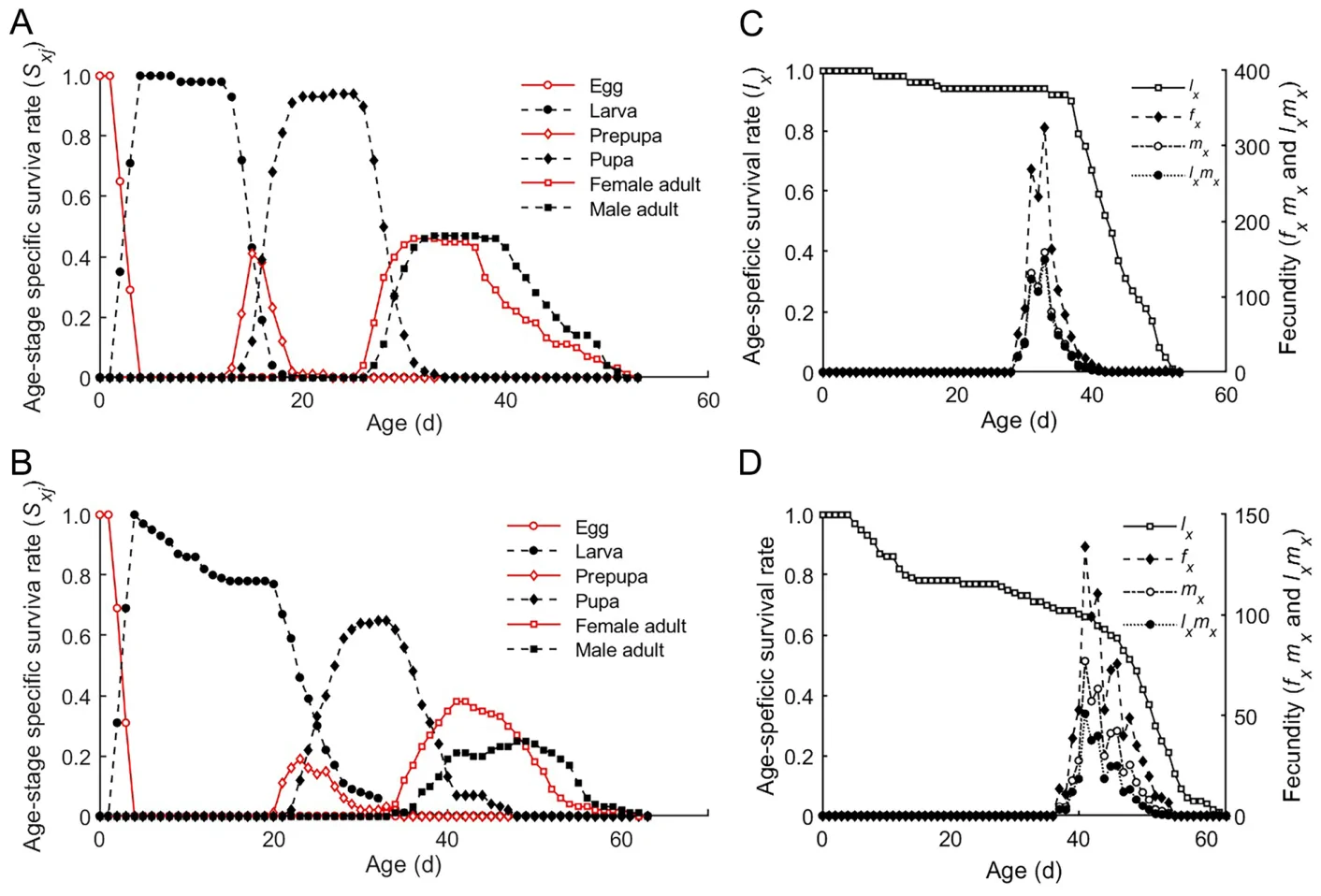
Age-stage specific survival rate (*s_xj_*) of FAW fed on Huang C (**A**) and CH1 (**B**), age-specific survival rate (*l_x_*), female age-specific fecundity (*f_x_*), age-specific fecundity of total population (*m_x_*), and age-specific maternity (*l_x_m_x_*) of FAW fed on Huang C (**C**) and CH1 (**D**).

Analysis of the *s_xj_*, *l_x_*, *f_x_*, *m_x_*, and *l_x_m_x_* curves revealed distinct impacts of the two maize germplasms on FAW survival and reproduction. For FAW reared on Huang C, the s_xj_ curve remained relatively stable across larval, pupal, and adult stages, accounting for 94.00% developing to the adult stage, while the CH1-fed group showed consistently declining larval, pupal, and adult survival with 65.00% developing to the adult stage (Figure 1A,B). The age-specific survival (*l_x_*) curve indicated high, stable survival for Huang C-fed FAW until day 37, followed by a rapid decline. In contrast, the CH1 group exhibited an early, rapid decline from day 5, with complex mortality dynamics thereafter. Furthermore, analysis of the *f_x_*, *m_x_*, and *l_x_m_x_* curves showed that while Huang C-fed FAW reached reproductive peak on day 33, the CH1-fed group peaked later on day 41 (Figure 1C,D).

### 3.3. Population Parameters of FAW on Two Maize Plants

Population parameters were calculated based on data from the entire cohort. The intrinsic rate of increase (*r*), finite rate of increase (*λ*), net reproductive rate (*R*_0_), and mean generation time (*T*) of FAW on two maize germplasms were calculated using the bootstrap method (Table 2). Results showed that the r, λ and *R*_0_ of FAW fed on CH1 were 0.1282, 1.1367 and 275.6000, which were respectively lower than those fed on Huang C. However, the *T*-value of FAW fed on CH1 (43.8403 d) was significantly longer than those fed on Huang C (33.4719 d).

### 3.4. Feeding and Oviposition Preference of FAW on Two Maize Plants

**Figure 2:**
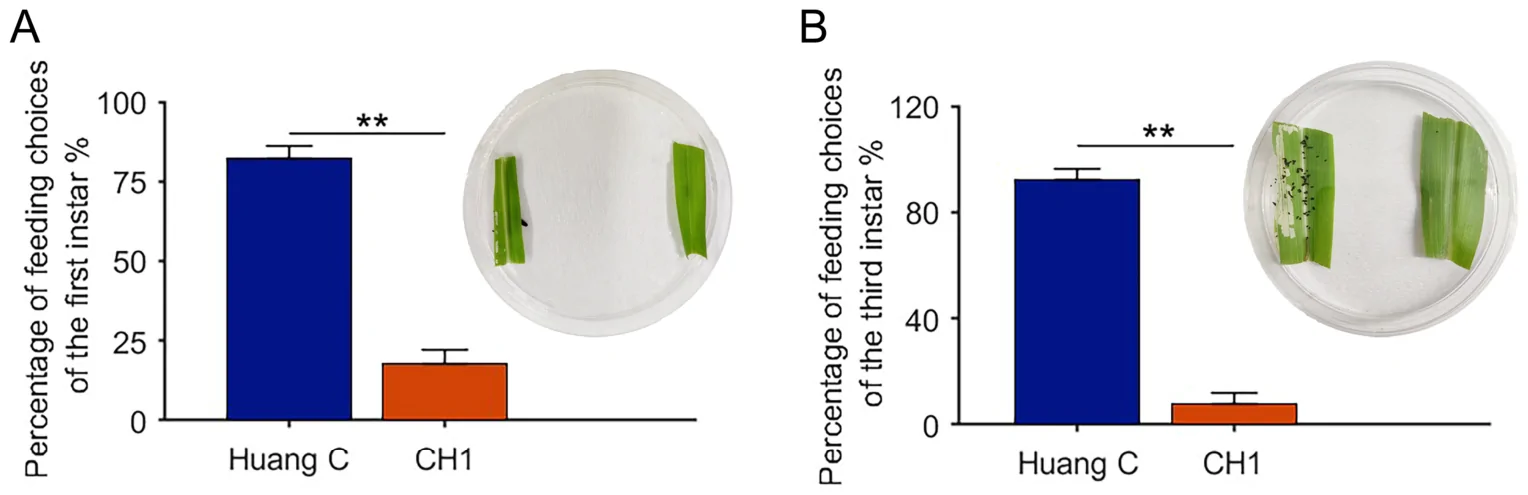
Feeding preference of choice assays. (**A**) Feeding preference of first-instar larvae. (**B**) Feeding preference of third-instar larvae. Data are expressed as mean ± SE. Asterisks ** indicate significant differences between Huang C and CH1 varieties at *p* < 0.01, as calculated by Student’s *t*-test.

**Figure 3:**
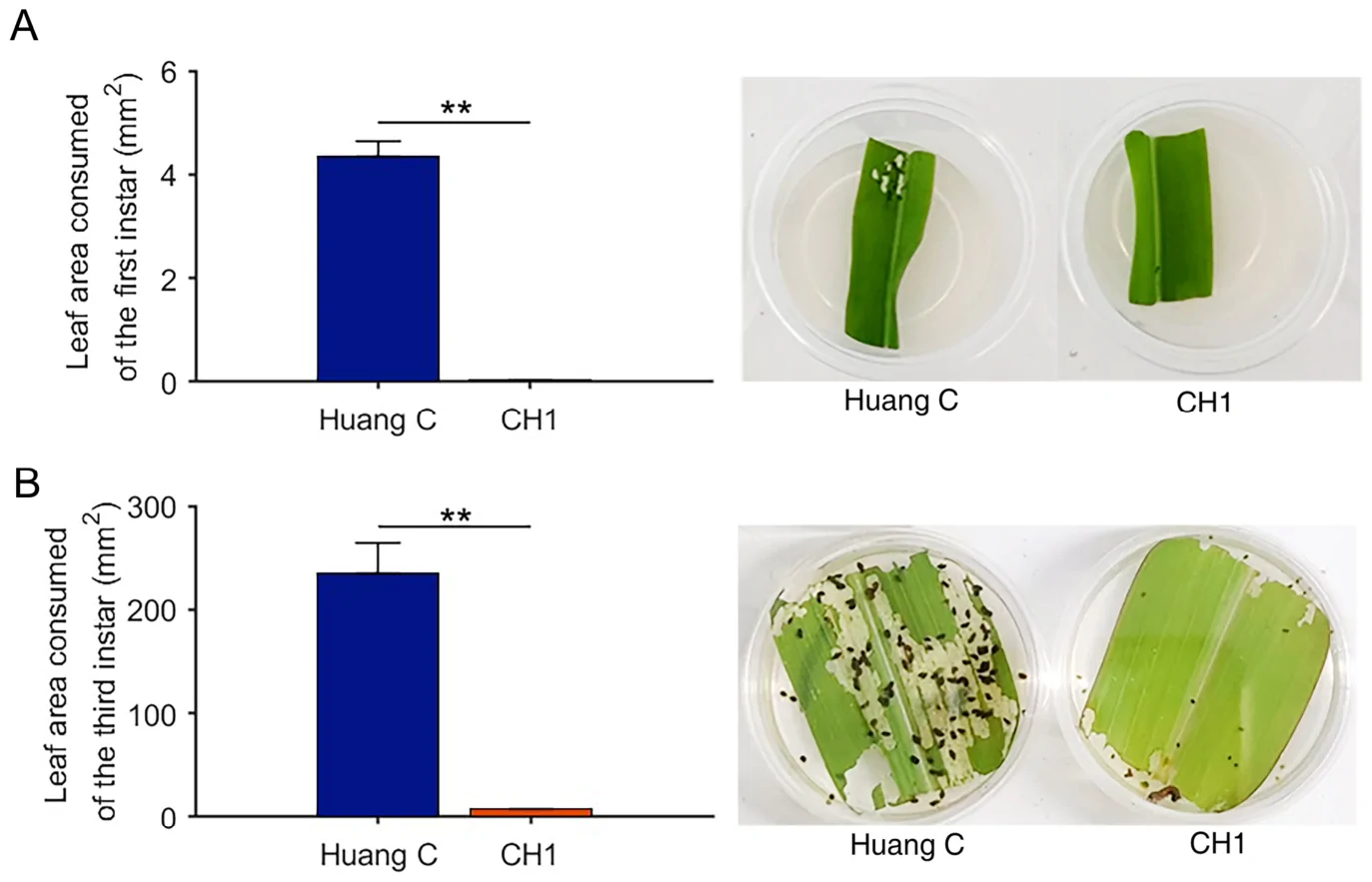
Leaf area consumed of no-choice assays. (**A**) Leaf area consumed by first-instar larvae. (**B**) Leaf area consumed by third-instar larvae. Data are expressed as mean ± SE. Asterisks ** indicate significant differences between Huang C and CH1 varieties at *p* < 0.01, as calculated by Student’s *t*-test.

In choice assays of feeding preference, both first- and third-instar larvae of FAW exhibited significantly higher feeding preference for Huang C than CH1 (Figure 2A,B; first-instar: *p* < 0.01; third-instar: *p* < 0.01). In no-choice assays of feeding preference, the leaf areas of Huang C consumed by first- and third-instar larvae of FAW were significantly larger than those of CH1 consumed by FAW (Figure 3A,B; first-instar: *p* < 0.01; third-instar: *p* < 0.01).

**Figure 4:**
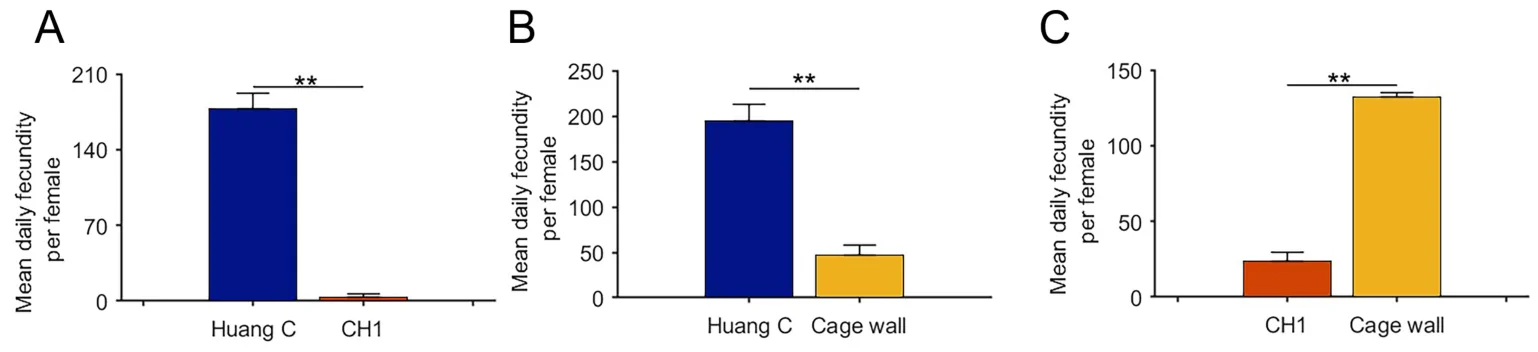
Oviposition preference of FAW on two maize germplasms. (**A**) Mean daily fecundity in Huang C and CH1. (**B**) Mean daily fecundity in Huang C and cage wall. (**C**) Mean daily fecundity in CH1 and cage wall. Data are expressed as mean ± SE. Asterisks ** indicate significant differences between Huang C and CH1 varieties at *p* < 0.01, as calculated by Student’s *t*-test.

In the choice assays of oviposition preference, FAW females significantly preferred to lay eggs on Huang C (177.89) over CH1 (3.33) (Figure 4A, *p* < 0.01). In the no-choice assays, females deposited eggs on both the maize plants and the cage walls. However, more eggs were laid directly on Huang C plants (194.50) than on the cage walls (47.50; *p* < 0.01), whereas the opposite pattern was observed for CH1, with more eggs being laid on the cage walls (132.25; *p* < 0.01) (Figure 4B,C).

### 3.5. Evaluation of Two Maize Germplasms’ Resistance to FAW

**Figure 5:**
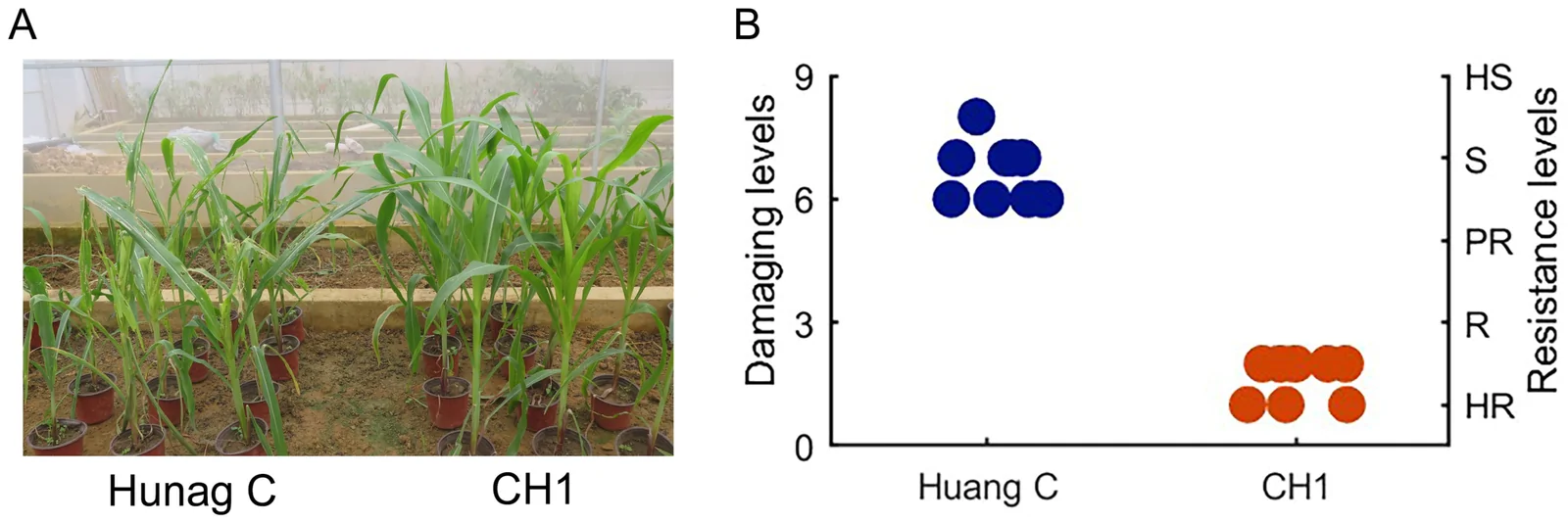
Photographs and resistance level of two maize cultivars from a gcreenhouse experiment. (**A**) Photograph from greenhouse experiment. (**B**) Damaging and resistance levels. HR stands for high resistance; R stands for resistance; PR stands for partially resistant; S stands for susceptibility; HS stands for high susceptibility.

As shown in Figure 5, plant damage was observed on Huang C, with its whorl and unfolded leaves exhibiting irregular feeding holes. In contrast, CH1 plants were largely undamaged, showing only minor pinhole-like feeding marks on a few older leaves (Figure 5A). According to the Davis scale for damage rating, Huang C scored between grades 6 and 8. The median damage score was seven, categorizing it as susceptible (S). In contrast, CH1 scored between one and two, with a median of two, demonstrating resistance (R) (Figure 5B).

## 4. Discussion

Developing and planting insect-resistant maize represents a central strategy in the integrated management of FAW, while identification and assessment of resistant germplasm are essential for breeding resistant varieties. In this study, we integrated life table analysis, behavioral preference assays and greenhouse experiment to evaluate the resistance of CH1 to FAW. Our results consistently indicate that the maize germplasm CH1 negatively affects FAW development, survival, reproduction, feeding preference, and oviposition preference, demonstrating resistance across multiple evaluation dimensions.

Host plants have significant effects on the development, survival, and reproduction of phytophagous insects. It is commonly asserted that short development duration, high survival rate, and large reproduction capacity of phytophagous insects represent high host fitness on certain plant species [22,33]. The age-stage, two-sex life table can reflect differences in the development rate between individuals and provide description of the performance of insect populations under experimental conditions [28,32,34]. In this study, FAW larvae reared on CH1 exhibited a significantly prolonged larval period, pre-pupal stage, a shortened adult lifespan and lower relative growth rate. Additionally, the *s_xj_* and *l_x_* curves further revealed markedly lower survival rates across all life stages for FAW on CH1, particularly during the early larval instars. This observed pattern suggests that CH1 may exert its strongest effects during the early larval establishment phase, as indicated by the rapid decline in the s_xj_ curve during the first-instar stages. However, statistical confirmation of stage-specific effects would require additional analyses (e.g., stage-specific survival comparisons) beyond the present dataset. More critically, FAW fed with CH1 had a lower population increase capacity (*r*, *R*_0_, *λ* were substantially lower, while *T* was longer). *R*_0_, *r*, *λ*, *T* are bio-parameter statistics that integrate insect growth and development, reproduction and survival changes and indicate the capacity for population growth in a specific environment [24]. A lower *R*_0_ value indicates fewer offspring produced per generation, and a reduced *r* value signifies a slower population growth potential [28]. The difference in key population growth parameters suggests that CH1 impairs FAW population growth and fitness, providing robust evidence for the antibiosis effect of CH1 on FAW. These findings align with previous studies on resistant host plants. For instance, the maize landrace Pérola extended the larval development of FAW and drastically reduced its larval “Fitness Index” [17]. Similarly, the unsuitable host plants were observed to have led prolonged development and decreased fecundity in FAW [35,36].

Many herbivorous insects can qualitatively distinguish among host plants or diets, preferentially feeding and ovipositing on high-quality plants [37]. In this study, FAW larvae significantly preferred to feed on Huang C than CH1, consuming a much larger leaf area on Huang C. Visually, the feeding damage on CH1 was minimal, often limited to small notches, whereas Huang C leaves suffered extensive defoliation. Additionally, in the oviposition choice tests, FAW females laid significantly more eggs on Huang C plants than on CH1 plants. In no-choice tests, females confined with CH1 laid the majority of their eggs on the cage walls rather than on the plants themselves. The preferences in oviposition selectivity showed that the females preferred Huang C over CH1, consistent with the preference performance hypothesis suggesting that females prefer to lay eggs on those hosts where larvae will thrive and actively avoid unsuitable hosts for egg-laying [30,31,32]. This finding is consistent with the study on potato tuber moth, where females also exhibited a clear preference for certain potato varieties over others, aligning with the subsequent performance of their offspring [38]. The results of feeding and oviposition deterrence are consistent with an antixenosis-type resistance mechanism in CH1, suggesting that CH1 may be an unfavorable host for FAW colonization and oviposition. However, the underlying mechanisms require further investigation.

FAW damage to maize germplasm is usually scored according to the Davis scale, which ranges from zero (no visible damage) to nine (whorl and furl leaves almost totally destroyed) based on leaf and whorl damage [30]. In our greenhouse experiment, CH1 plants sustained minimal damage (Davis scale grade 2—resistant), whereas Huang C plants were severely damaged (grade 7—susceptible). Previous studies reported that most native or naturally occurring resistance is polygenic, exhibits partial resistance, and scores around 3–5 on the Davis scale, whereas transgenic lines exhibit scores of 1–2 [15]. As a native germplasm, CH1 exhibits score of 2, representing a promising genetic resource for developing FAW-resistant varieties, subject to further validation under field conditions.

Taken together, our results demonstrate that CH1 negatively affects FAW’s development, survival, reproduction, feeding preference, and oviposition preference. Greenhouse experiment suggests that CH1 exhibits high resistance to FAW larvae. These findings identify CH1 as a promising resistant germplasm that warrants further evaluation in field settings and breeding programs. However, field validation is necessary before conclusions about CH1 representing a highly resistant germplasm for practical FAW management can be made. Before CH1 can be recommended for commercial cultivation, additional agronomic evaluations are necessary. Future studies should compare CH1 with currently cultivated maize varieties in China with respect to key agronomic traits, including grain yield, kernel quality, maturity period, lodging resistance, and drought tolerance. The cost-effectiveness of CH1 seeds and the potential trade-offs between pest resistance and yield performance should also be assessed under field conditions. These evaluations will determine the practical feasibility of incorporating CH1 into integrated pest management (IPM) programs for FAW in China.

## Figures and Tables

**Table 1:** Development duration, pupal weight, adult longevity and reproduction of FAW fed on two maize leaves.

Parameter	Huang C	CH1	*p*-Value
Egg (d)	2.94 ± 0.08	3.00 ± 0.08	*p* = 0.86
Larva (d)	12.49 ± 0.10	21.61 ± 0.36	*p* < 0.01 **
Pre-pupa (d)	1.49 ± 0.05	1.71 ± 0.06	*p* < 0.01 **
Pupa (d)	11.86 ± 0.09	12.37 ± 0.16	*p* = 0.99
Adult (d)	14.96 ± 0.43	13.24 ± 0.47	*p* < 0.01 **
Female pupal weight (g)	0.16 ± 0.0022	0.15 ± 0.0028	*p* = 0.99
Male pupal weight (g)	0.16 ± 0.0026	0.17 ± 0.0043	*p* = 0.21
Female longevity (d)	14.81 ± 0.46	12.89 ± 0.51	*p* = 0.04 *
Male longevity (d)	15.42 ± 0.59	13.89 ± 0.86	*p* = 0.14
Pre-oviposition (d)	3.24 ± 0.17	4.38 ± 0.26	*p* < 0.01 **
Total pre-oviposition (d)	31.22 ± 0.20	41.77 ± 0.37	*p* < 0.01 **
Oviposition period (d)	8.52 ± 0.35	6.62 ± 0.36	*p* < 0.01 **
Fecundity (eggs/female)	1368.93 ± 40.62	689.00 ± 39.09	*p* < 0.01 **

Data are mean ± SE. Asterisks (*) and (**) indicate significant differences between Huang C and CH1 varieties at *p* < 0.05 and *p* < 0.01, respectively, as calculated by Student’s *t*-test. The sample sizes for pupal weight measurements were as follows: female pupae: *n* = 46 (Huang C) and *n* = 42 (CH1); male pupae: *n* = 49 (Huang C) and *n* = 30 (CH1).

**Table 2:** Population parameters of FAW fed on two maize plants.

Parameter	HuangC	CH1	*p*-Value
Intrinsic rate of increase (*r*)	0.1926 ± 0.0037	0.1282 ± 0.0034	*p* < 0.01 **
Finite rate of increase (*λ*)	1.2123 ± 0.0044	1.1367 ± 0.0038	*p* < 0.01 **
Net reproductive rate (*R*_0_)	629.7100 ± 70.8959	275.6000 ± 37.0116	*p* < 0.01 **
Mean generation time (*T*)	33.4719 ± 0.1798	43.8403 ± 0.3624	*p* < 0.01 **

Data are mean ± SE. Asterisks (**) indicate significant differences between Huang C and CH1 varieties at *p* < 0.01, respectively, as calculated by paired bootstrap test.

## Data Availability

The data that support the findings of this study are available from the corresponding authors, upon reasonable request.
